# The Use of Motion-Based Technology for People Living With Dementia or Mild Cognitive Impairment: A Literature Review

**DOI:** 10.2196/jmir.6518

**Published:** 2017-01-11

**Authors:** Erica Dove, Arlene J Astell

**Affiliations:** ^1^ Research & Academics Ontario Shores Centre for Mental Health Sciences Whitby, ON Canada; ^2^ Rehabilitation Sciences Institute University of Toronto Toronto, ON Canada

**Keywords:** dementia, mild cognitive impairment, technology, review

## Abstract

**Background:**

The number of people living with dementia and mild cognitive impairment (MCI) is increasing substantially. Although there are many research efforts directed toward the prevention and treatment of dementia and MCI, it is also important to learn more about supporting people to live well with dementia or MCI through cognitive, physical, and leisure means. While past research suggests that technology can be used to support positive aging for people with dementia or MCI, the use of motion-based technology has not been thoroughly explored with this population.

**Objective:**

The aim of this study was to identify and synthesize the current literature involving the use of motion-based technology for people living with dementia or MCI by identifying themes while noting areas requiring further research.

**Methods:**

A systematic review of studies involving the use of motion-based technology for human participants living with dementia or MCI was conducted.

**Results:**

A total of 31 articles met the inclusion criteria. Five questions are addressed concerning (1) context of use; (2) population included (ie, dementia, MCI, or both); (3) hardware and software selection; (4) use of motion-based technology in a group or individual setting; and (5) details about the introduction, teaching, and support methods applied when using the motion-based technology with people living with dementia or MCI.

**Conclusions:**

The findings of this review confirm the potential of motion-based technology to improve the lives of people living with dementia or MCI. The use of this technology also spans across several contexts including cognitive, physical, and leisure; all of which support multidimensional well-being. The literature provides evidence that people living with dementia or MCI can learn how to use this technology and that they enjoy doing so. However, there is a lack of information provided in the literature regarding the introduction, training, and support methods applied when using this form of technology with this population. Future research should address the appropriate introduction, teaching, and support required for people living with dementia or MCI to use the motion-based technology. In addition, it is recommended that the diverse needs of these specific end-users be considered in the design and development of this technology.

## Introduction

Dementia is a collective term used to describe a range of symptoms associated with neurodegenerative conditions such as Alzheimer’s disease. Characteristic signs of dementia involve marked impairment in the areas of cognitive functioning such as memory, attention, executive function, comprehension, judgment, and communication [1]. Mild cognitive impairment (MCI) can be defined as a condition in which an individual has mild but measurable changes in thinking abilities; however, these changes do not affect the person’s ability to carry out all activities of daily living [2]. MCI can be classified as amnestic or nonamnestic based on the thinking abilities affected [2]. For example, people with amnestic MCI predominantly experience memory challenges and may begin to forget important information that he or she would have previously recalled easily (eg, appointments, conversations, or recent events). In contrast, memory is spared among people with nonamnestic MCI, with changes greater than those expected for their age in one other cognitive domain such as language, visuospatial skills, or executive functioning [2]. As the population ages and life expectancy continues to increase [3], progressive cognitive disorders such as dementia or MCI have become a major age-related health concern, with the number of people living with dementia expected to reach 1.4 million in Canada by 2031 [4]. Although there are many research efforts directed toward the prevention and treatment of dementia and MCI, it is also important to learn more about supporting a good life for people currently living with these conditions [5].

People living with dementia or MCI often have reduced opportunities for engagement and face progressive barriers when trying to participate in activities they once enjoyed with ease [5]. For individuals with dementia or MCI, engaging in pleasant and meaningful activities can promote a good quality of life and support overall well-being [6]. Recent publications have shown that there are many ways in which people with dementia can engage in pleasant activities and live well, including the use of novel technology devices such as touchscreen tablets and personal computers [5,7-10].

More recently, the use of motion-based technology has become a popular topic of interest in many areas of care [11-16]. Recent research involving the motion-based technology has revealed positive benefits for healthy older adults [11-13], as well as populations with stroke [14], Parkinson’s disease [15], and traumatic brain injury [16]. Motion-based technology can be described as a natural user interface (NUI) device that operates through intuitive actions similar to natural, everyday gestures. When used for activity promotion, games presented through motion-based technology may also be referred to as “exergames” as physical actions are required to interact with the technology and play the games. In addition, games presented on this type of technology can provide an accessible source of fun and meaningful engagement. However, the literature on the use of motion-based technology for people living with dementia or MCI is currently quite limited and undefined. Therefore, the purpose of this literature review is to identify and synthesize the current knowledge on this topic, while highlighting gaps that require further investigation. In addition, this review aims to add to the body of knowledge supporting the use of novel technologies to promote a good life for people living with dementia or MCI.

The current review presents an overview of the ways in which motion-based technology has been used with people living with dementia or MCI. This review aims to address the following questions:

1. In what contexts has motion-based technology been used with people living with dementia or MCI?

2. Were these studies focused on people with dementia or people with MCI?

3. What forms of hardware and software were used?

4. Was the technology utilized in a group or individual setting?

5. What methods were used to introduce, teach, and support people living with dementia or MCI to use motion-based technology?

## Methods

A systematic review of the literature was conducted on the use of motion-based technology for people living with dementia or MCI.

The following search terms were used for this review: (dementia) OR (Alzheimer*) OR (mild cognitive impairment) OR (MCI) AND (exergam*) OR (motion-based) OR (virtual reality) OR (gesture-based) OR (Nintendo Wii) OR (Xbox Kinect) OR (interactive console) AND (activit*) OR (gam*).

The following electronic databases were accessed for this review: Medline (Ovid), PsycINFO (Ovid), Cochrane (Ovid), PubMed (NCBI), and CINAHL (EBSCO). The search was extended to include references of relevant articles.

Articles were included or excluded based on the following criteria: (1) language: English; (2) participants: human, living with dementia or MCI; and (3) technology: any interface requiring physical movement for interaction.

**Figure 1:**
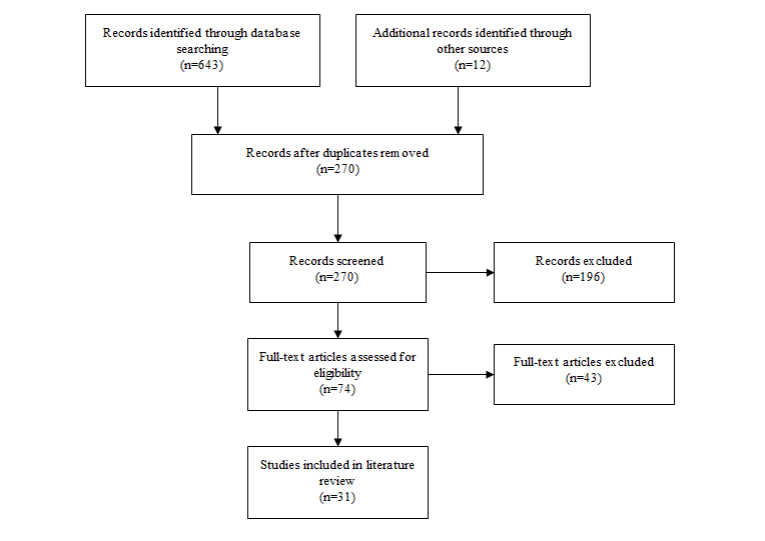
Flow diagram of search procedure.

The search protocol defined above originally resulted in the identification of 643 references through database searching and an additional 12 references through hand searching, amounting to a total of 655 initial returns. After the removal of duplicate documents, a total of 270 articles remained which were then screened for eligibility criteria. Articles that did not meet the inclusion criteria based on their title or abstract were subsequently removed, resulting in a total of 74 articles identified for full-text analysis. After reading each article, an additional 43 articles were excluded, resulting in a total of 31 full-text articles included in the final review (Figure 1) [17].

## Results

### Overview of Findings

Thirty-one articles met the final inclusion criteria and were included in this review. Table 1 shows the synthesized results of the search procedure that provides an overview on this topic, organized as per the questions identified above.

### Context of Use

Three broad categories describing the context in which the motion-based technology was used for people living with dementia or MCI were identified during the review: (1) cognitive function (21 articles); (2) physical function or activity promotion (12 articles); and (3) leisure activities (13 articles). Thirteen articles contained information pertaining to one or more categories that suggests the motion-based technology has the potential to span across several contexts. Articles addressing more than one context of use include: 6 articles [21,24,32,34,46,47] focused on cognitive function and leisure activities, 3 articles [23,37,38] pertaining to both cognitive and physical function, 2 articles [30,44] relevant to both physical function and leisure activities, and 2 articles [26,45] encompassing all 3 categories. These articles were counted under both or all categories as appropriate (Table 1).

#### Cognitive Function (n=21)

Cognitive function was the most prevalent category identified within the literature regarding the use of motion-based technology for people with dementia or MCI. Articles categorized in this section used the motion-based technology to present cognitive exercises to people with dementia or MCI with the aim of assessing, measuring, restoring, maintaining, stimulating, or improving various aspects of cognitive function. The articles included in this section used a technology that is commercially available (eg, Nintendo Wii) [24,26,28,31,32,34- 38,45-47], or the one that was specifically created for study purposes by the researcher (eg, a stationary bicycle merged with a graphical interface) [18,21-23,27,29,43,48].

The main focus was general cognitive function rather than focusing on specific domains (eg, memory, executive function) [18,24,29,31,45,46,48]. Articles included in this category used the motion-based technology with the aim of assessment and screening for early signs of dementia in people living with MCI [37,43]; reducing the risk or rate of progression in people currently living with dementia [21,23,32,38]; assessing or improving procedural learning ability in people with either dementia or MCI [28,34,35]; providing cognitive stimulation for people with dementia [26-28]; prompting people with dementia through occupational tasks [22]; increasing the attention span and concentration during tasks for people with MCI [47]; and improving reaction time (ie, how fast the brain responds to stimuli) and comprehension in people with MCI [36].

**Table 1:** Summarized results of literature review.

Publication author	Purpose of technology	Study population	Hardware and software	Individual or group use	Introduction, teaching, and support methods used
Bamidis et al [18]	Cognitive function	Dementia and MCI^a^	Touchscreen interface, Nintendo Wii-mote, and Wii Balance Board (FitforAll); SketchUp (Google, Mountain View), 3Ds Max Studio (Autodesk Inc), and XNA game software (Microsoft Corp)	Group	-
Benveniste et al [19]	Leisure activities	Dementia	TV, PC, sensor bar, and Nintendo Wii-mote (MinWii); unspecified software	Group	Staff would sit with participants and offer as much support as needed
Billis et al [20]	Physical function or activity promotion	Dementia and MCI	Touchscreen interface, Nintendo Wii-mote and Wii Balance Board (FitforAll); SketchUp (Google, Mountain View), 3Ds Max Studio (Autodesk Inc), and XNA game software (Microsoft Corp)	Group	Psychologist or therapist was present during all sessions to train the participants in using the program
Boulay et al [21]	Cognitive function and leisure activities	Dementia	TV, PC, sensor bar, and Nintendo Wii-mote (MinWii); unspecified software	Group	Participants were given 1 training session to become familiar with the interface; psychologists gave instructions and help during test sessions
Chang et al [22]	Cognitive function	Dementia	Microsoft Kinect sensor, PC, and TV screen; Kinempt software (custom-built)	Individual	Verbal instructions, task breakdown, gesture demonstrations, support as required
Chilukoti et al [23]	Cognitive function and physical function or activity promotion	Dementia	Mini stationary bike with graphical user interface; unspecified software	Individual	-
Colombo et al [24]	Cognitive function and leisure activities	Dementia and MCI	EyeToy for PlayStation 2; Bubblepop game (Sony Corp)	Individual	-
Cutler et al [25]	Leisure activities	Dementia	Nintendo Wii; Wii Sports, and Nintendo Wii Fit (Nintendo Co Ltd)	Group	Repeated demonstrations
Cutler et al [26]	Cognitive function, physical function or activity promotion, and leisure activities	Dementia	Nintendo Wii; Wii Fit (Nintendo Co Ltd,), and Microsoft Kinect; Kinect Sports (Microsoft Corp)	Group	Initial support, reduced as participants became competent with the technology
De Urturi Breton et al [27]	Cognitive function	Dementia	Microsoft Kinect; KiMentia software (custom-built)	Individual	-
Fenney and Lee [28]	Cognitive function	Dementia	Nintendo Wii; Nintendo Wii Sports (Nintendo Co Ltd)	Group	Physical guidance, verbal prompts as required
González-Palau et al [29]	Cognitive function	MCI	Touchscreen interface, Nintendo Wii-mote, and Wii Balance Board (FitforAll); SketchUp (Google, Mountain View), 3Ds Max Studio (Autodesk Inc), and XNA game software (Microsoft Corp)	Individual	Participant met with a therapist every 2 weeks to discuss progress
Higgins et al [30]	Physical function or activity promotion and leisure activities	Dementia and MCI	Nintendo Wii; Nintendo Wii Sports (Nintendo Co Ltd)	Group and individual	Introduced in a one-on-one session before transitioning to a group environment
Hughes et al [31]	Cognitive function	MCI	Nintendo Wii; Nintendo Wii Sports (Nintendo Co Ltd)	Group	Initial training for 6 weeks on how to use the technology
Kayali et al [32]	Cognitive function and leisure activities	Dementia	Nintendo Wii; Nintendo Wii Fit (Nintendo Co Ltd)	Group	-
Konstantinidis et al [33]	Physical function or activity promotion	Dementia	Touchscreen interface, Nintendo Wii-mote, and Wii Balance Board (FitforAll); Google SketchUp (Google, Mountain View), 3Ds Max Studio (Autodesk Inc), and XNA game software (Microsoft Corp)	Individual	Technology gives instructions, prompts, praise, notifications, and guidance; therapists can add or modify games
Leahey and Singleton [34]	Cognitive function and leisure activities	Dementia	Nintendo Wii; Nintendo Wii Sports (Nintendo Co Ltd)	Group	Verbal and physical cues, vanishing cues, task breakdown
Legouverneur et al [35]	Cognitive function	Dementia and MCI	Nintendo Wii; Nintendo Wii Sports (Nintendo Co Ltd)	Individual	Simple introduction, repeated demonstrations, verbal prompts, physical assistance when required
Liou et al [36]	Cognitive function	MCI	Microsoft Kinect; Kinect Sports, Kinectimals and Fruit Ninja (Microsoft Corp)	Group	-
McCallum and Boletsis [37]	Cognitive function and physical function or activity promotion	Dementia and MCI	Nintendo Wii; Nintendo Wii Fit, and Nintendo Wii Sports (Nintendo Co Ltd)	Group and individual	-
McCallum and Boletsis [38]	Cognitive function and Physical function or activity promotion	Dementia and MCI	Nintendo Wii; Nintendo Wii Fit and Nintendo Wii Sports (Nintendo Co Ltd)	Group and individual	-
McEwen et al [39]	Physical function or activity promotion	Dementia	Interactive rehabilitation exercise (IREX) hardware and software (GestureTek, Silicon Valley)	Individual	-
Padala et al [40]	Physical function or activity promotion	MCI	Nintendo Wii; Nintendo Wii Fit (Nintendo Co Ltd)	Individual	-
Padala et al [41]	Physical function or activity promotion	MCI	Nintendo Wii; Nintendo Wii Fit (Nintendo Co Ltd)	Individual	-
Siriaraya and Ang [42]	Leisure activities	Dementia	Microsoft Kinect; software created through Unity3D (Unity Technologies)	Group and individual	Prompts provided as required based on each person
Tarnanas et al [43]	Cognitive function	Dementia and MCI	PC, Microsoft Kinect sensor, treadmill and LEAP motion sensor (Leap Motion Inc); Microsoft Kinect software development kit (Microsoft Corp)	Individual	-
Tobiasson [44]	Physical function or activity promotion and leisure activities	Dementia	Nintendo Wii; Nintendo Wii Sports (Nintendo Co Ltd)	Group	Gesture demonstrations, verbal cues, physical support when required, personalizing introduction, teaching, and support methods
Tobiasson et al [45]	Cognitive function, physical function or activity promotion and leisure activities	Dementia	Nintendo Wii; Nintendo Wii Sports (Nintendo Co Ltd)	Group	Initial support, reduced over time as users became more competent with the technology
Ulbrecht et al [46]	Cognitive function and leisure activities	Dementia and MCI	Nintendo Wii; Nintendo Wii Sports (Nintendo Co Ltd)	Group and individual	Therapists received training in introducing and supervising the intervention prior to the study
Weybright et al [47]	Cognitive function and leisure activities	MCI	Nintendo Wii; Nintendo Wii Sports (Nintendo Co Ltd)	Individual	One-on-one introduction session to learn the technology and play the games; prompts and cues provided as needed prior to and during the sessions
Yamaguchi et al [48]	Cognitive function	Dementia	XaviX hardware—base-machine, TV screen, sensor mat and sensor ball; Hot-plus software (SSD Co Ltd)	Group	Caregivers support participants through communication and praise

^a^MCI: Mild cognitive impairment.

Whereas there were many different ways in which motion-based technology was applied as a cognitive intervention for people living with dementia or MCI, the rationales behind the choice to use this technology were quite similar. Many studies chose to use motion-based technology as evidence supports combined mental and physical training for cognitive health promotion [18,23,27,29,32,34,36,38,45-47]. This type of technology was also chosen due to the fact that it provides cognitive [21,24,26,28,35], mental and social [31,48], and physical and social [37] stimulation for people living with dementia or MCI. In additional, motion-based technology is intuitive and has the unique ability to perform tasks such as assessment and screening and task prompting for individuals with dementia or MCI [22,43].

#### Physical Function or Activity Promotion (n=12)

Physical function or activity promotion was the category least focused on in respect to people living with dementia or MCI. Articles included in this category [20,23,26,30,33,37-41,44,45] used the motion-based technology to target areas of physical functioning, remove or reduce risk of disability, provide a targeted rehabilitation approach for those already living with existing physical challenges, and promote games presented on the motion-based technology as an engaging, attainable, and accessible way to encourage people living with dementia or MCI to participate in physical activity. Articles included within this context also featured commercially available technology [26,30,37,38,40,41,44,45] and technology designed by researchers specifically for study purposes [20,23,33,39].

One common theme to emerge was gait, balance, and fall risk [37-41]. Additional areas of focus within this emerging theme included general motor function [37,38] and mobility in people with either dementia or MCI [30,39], as these measures can be indicative of an increased risk for falls. The literature also placed a great deal of focus on using the motion-based technology as an interactive, accessible, and fun way to encourage motivation to participate in and enjoy physical activity in general [20,23,26,30,33,44,45]. People with dementia or MCI face many challenges that can make it increasingly difficult to engage in regular physical activity or rehabilitation regimens. Unsurprisingly, this technology was mainly utilized as a tool for activity promotion.

#### Leisure Activities (n=13)

Leisure activities can be described as fun and enjoyable pastimes that contribute to a higher quality of life [5]. In this review, it was found that only 42% (13/31) studies used the motion-based technology to provide meaningful leisure activities for people with dementia or MCI [19,21,24-26,30,32,34,42,44-47] compared with the 68% (21/31) studies that used the motion-based technology for cognitive purposes [18,21-24,26-29,31,32,34- 38,43,45-48]. Interestingly, a review of the use of touchscreen technology for people living with dementia [9] highlighted that supporting leisure activities has received significantly less focus compared with the context of cognitive function. This is echoed in the current review of the motion-based technology, which has been used more as a cognitive tool than a leisure device. Indeed, there are areas of the leisure technology industry dedicated to this, but their emphasis has tended to be on the younger generations as their target consumers [49]. For example, it has been well established that compared with younger people, older adults have lower access to and usage of recent technologies [49].

The articles within the leisure category used the motion-based technology with the primary aim of providing a source of enjoyment and engagement for people living with dementia or MCI [19,25,26,30,32,34,42,44,45]. In addition, the articles examined the use of the motion-based technology for other aspects of leisure such as acceptability and pleasure of games presented on the motion-based technology for people with dementia or MCI [24,46], self-esteem in people with dementia [19,21], positive affect and mood in people with MCI [47], and reminiscing in people with dementia [42]. Interestingly, in many of the articles that did not address leisure, it was still reported that the participant dropout rate was relatively low, and that they enjoyed interacting with the technology and playing the games. This suggests that the motion-based technology has the ability to provide an inadvertent source of enjoyment, even if it is technically being utilized as a cognitive or physical tool.

Two of the reviewed articles in the leisure category described “gaming technology groups” specifically for people with dementia that aimed to use games presented on motion-based technology to provide cognitive and physical stimulation, as well as meaningful leisure activities within an interactive social setting [25,26]. The authors ran these group activities with various subsets of the target population including people living with dementia within the community, people living in assisted-living facilities, male-only groups, and groups combining men and women. Investigators behind the “gaming groups” used the knowledge acquired to create “how to” guides for implementing, managing, and supporting gaming technology groups for people living with dementia [50,51].

### Study Population

The second research question, which aims to identify the study population included in the interventions (ie, people with dementia or people with MCI) have been discussed here. Of note is that 9 of the 31 articles included in this review focused on both people with dementia and people with MCI [18,20,24,30,35,37,38,43,46]; therefore, these studies have been counted under both categories.

#### People With Dementia (n=25)

Of the 31 studies included in the review, 25 of them focused on people with dementia. This shows a clear trend toward the use of the motion-based technology for people with dementia rather than people with MCI. In addition, it is unsurprising that the category of cognitive function received the greatest attention in studies involving people with dementia, with 17 of 25 articles addressing an aspect of cognition.

Of interest, a majority of the studies involving people with dementia were conducted in a group setting, whereas those focusing on people with MCI were conducted as an individual activity. The reasoning behind this was not clear. In addition, 16 of the 25 articles focusing on people with dementia conveyed information regarding the methods used to introduce, teach, and support people with dementia to use the motion-based technology. In contrast, only 7 of 15 articles focused on the use of the motion-based technology for people with MCI offered all the information regarding the introduction, teaching, and support methods. Again, the reasoning behind this was not exactly clear. Finally, studies looking at these 2 groups, whether together or separately, all chose to utilize more commercially available hardware and software than custom-built.

#### People With Mild Cognitive Impairment (n=15)

Of the 31 studies included in this review, only 15 focused on people living with MCI. Of note is that none of these articles specified whether participants had amnestic or nonamnestic MCI. Furthermore, only 6 of these 15 studies focused specifically on people with MCI, with the remainder focusing on people with MCI in conjunction with people living with dementia. This highlights the lack of focus on people living with MCI specifically within the motion-based technology literature.

In studies looking specifically at people with MCI, cognitive function also received the most attention, similar to the studies focusing on people with dementia. However, there was much less focus on the use of the motion-based technology to provide meaningful leisure activities for people with MCI (27%, 4/15 studies) than there was for people with dementia (48%, 12/25 studies). In addition, the number of studies looking at physical function or activity promotion for people with MCI was higher than those looking at physical function or activity promotion in people with dementia. This indicates that interventions for people with MCI specifically were used more for improving cognition or physical function, whereas studies involving people with dementia specifically tended to focus more on cognition and leisure, with physical function or activity promotion receiving the least amount of focus.

### Hardware and Software

The hardware (ie, console used) and software (ie, gaming interface) utilized in the review articles have been discussed here. Of note is that multiple articles within the cognitive function, leisure activities, and physical function or activity promotion contexts all used similar hardware and software (Table 1), suggesting that the existing motion-based technology has the ability to span across several contexts.

#### Hardware Selection

**Figure 2:**
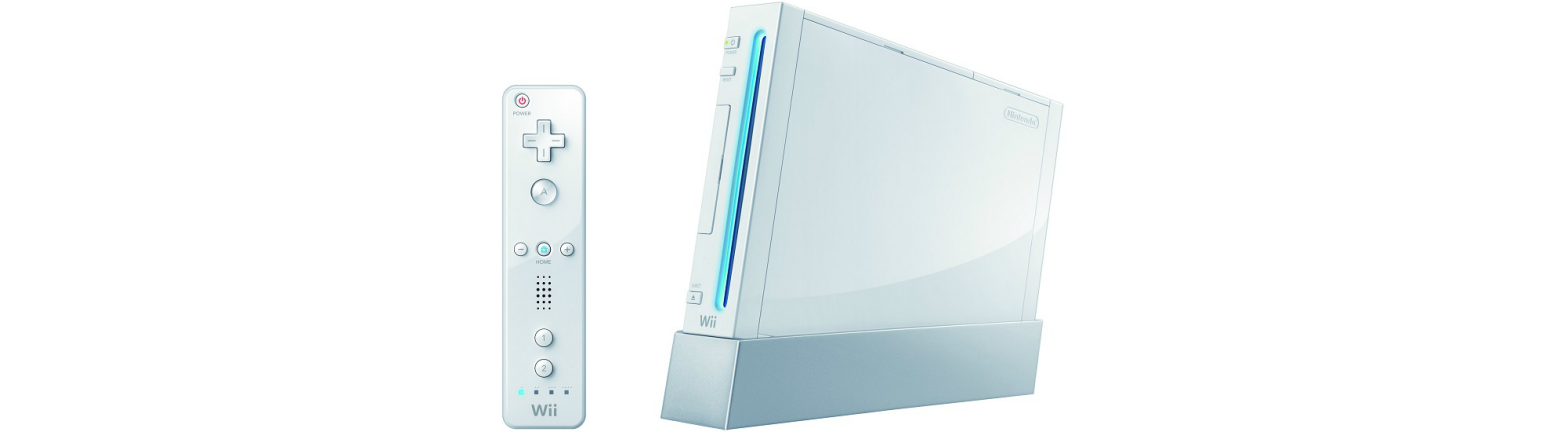
Nintendo Wii controller (left) and Nintendo Wii base console (right).

**Figure 3:**
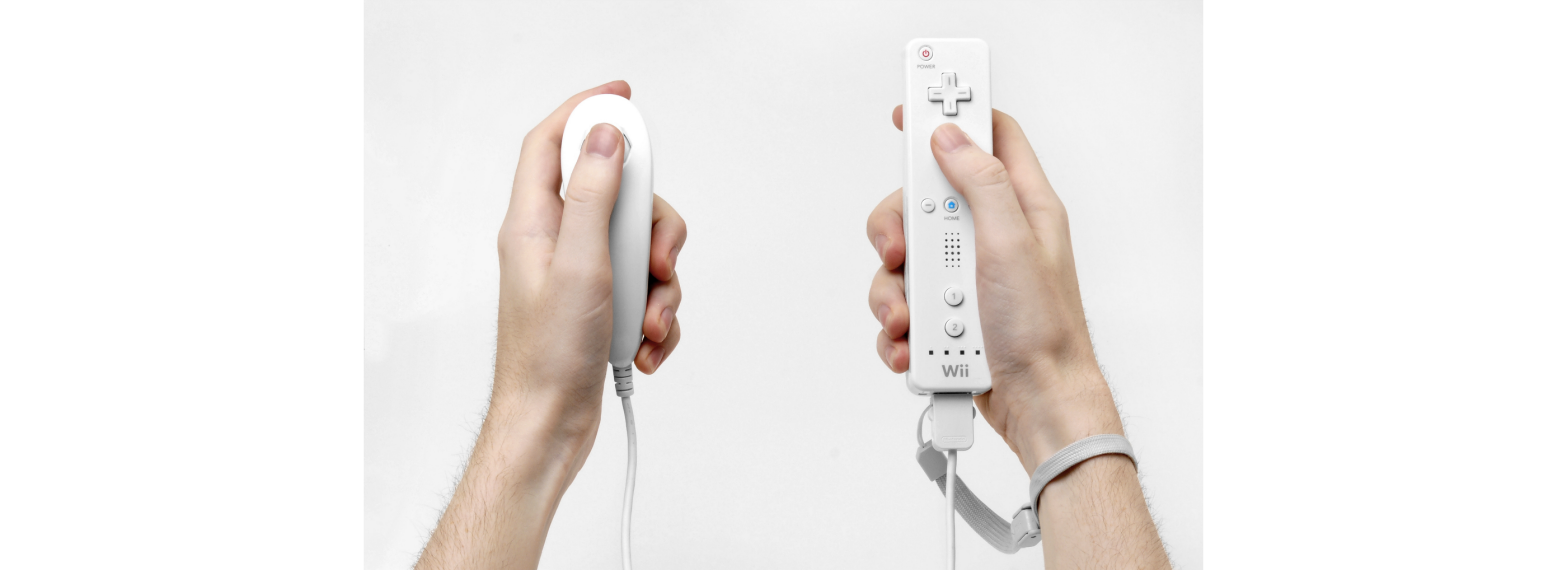
Nintendo Wii interaction.

An analysis of the literature revealed that a majority of the research involving the motion-based technology and people with dementia or MCI utilized commercially available hardware. The most commonly chosen motion-based technology hardware, used in 16 studies [25,26,28,30-32,34,35,37,38,40,41,44-47], was the Nintendo Wii (Nintendo Co Ltd), which features a base console and a remote control with built-in motion sensors. The player holds the remote control (also known as the “Wii-mote”) while performing physical movements, which are transformed into actions on the screen (Figures 2 and 3). However, holding and operating the Wii-mote requires manual dexterity, fine motor control, grip strength, and may be difficult to use for populations with age-related physical impairments, as well as people with dementia or MCI. In one instance, Tobiasson et al, 2015 [45] had to modify the Wii-mote by covering most of the buttons with a thermoplastic splint to make it easier to hold and prevent players with dementia from unintentionally pressing the wrong buttons or pressing too many buttons. This was echoed in studies by Benveniste et al, 2010 [19] and Boulay et al, 2011 [21], where subjects used a Wii-pistol (a small plastic gun that holds the Wii-mote) to make holding and using the controller easier. In addition, participants with dementia or MCI both reported having difficulty remembering which buttons on the Wii-mote were associated with which movements, therefore, this issue was mitigated by covering most of the buttons [19,21,30,45].

**Figure 4:**
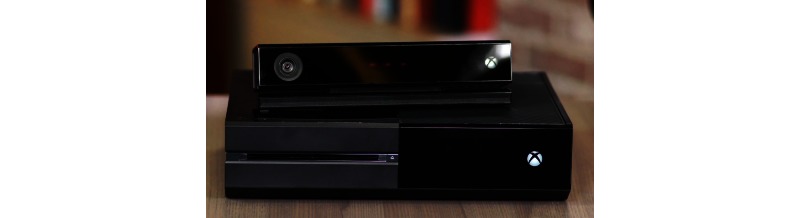
Xbox One base console (bottom) and Microsoft Kinect sensor (top).

**Figure 5:**
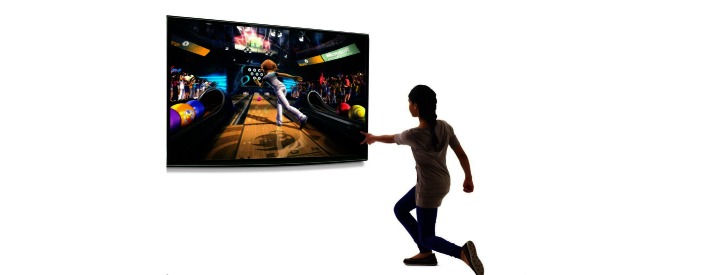
Microsoft Kinect interaction.

The Xbox Kinect (Microsoft Corp) was the second most common commercially available hardware device featured within the literature [26,27,36,42]. The Kinect sensor, used in conjunction with a base console, is embedded with gesture-recognition equipment including an infrared projector and a camera that tracks motion in 3 different dimensions. Movements made by the player are mimicked by a virtual avatar on the screen. Due to the fact that there is no handheld remote or controller required, the user controls the entire gaming interaction through the use of physical motions, allowing for an immersive and user-friendly experience (Figures 4 and 5).

**Figure 6:**
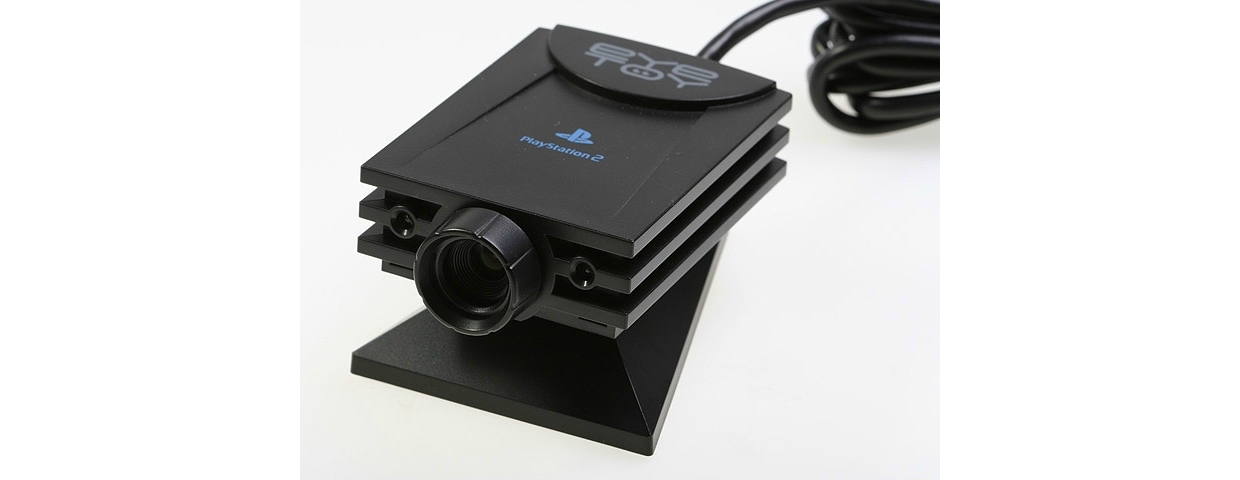
PlayStation2 EyeToy hardware.

**Figure 7:**
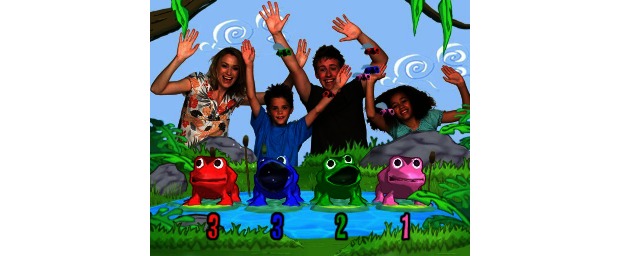
PlayStation2 EyeToy interaction.

Colombo et al’s study (2012) [24] was the only one to utilize the EyeToy for PlayStation2 hardware (Sony Corp) that features a small USB camera connected to the main console that displays the image of the person in view on the TV screen. Similar to the Microsoft Kinect, interaction requires no handheld controller, allowing the user to naturally interact with the game (Figures 6 and 7). However, participants’ perceptions of the usability or acceptability of this hardware was not mentioned in the article, and there was no other mention of this specific device within any of the other articles.

About eleven articles featured custom-built hardware [18-23,29,33, 39,43,48]. The hardware designs varied, including a personal computer merged with a Microsoft Kinect sensor and a TV screen [22]; a mini stationary bike with an attached graphical user interface [23]; a TV screen merged with a PC, a sensor bar, and a Wii-mote controller [19,21]; a personal computer merged with a Microsoft Kinect sensor, a treadmill, and a LEAP sensor (Leap Motion Inc) [43]; a base-machine combined with a television screen, a sensor mat, and motion-sensor wrist bands [48]; green screen hardware [39]; and a touchscreen interface compatible with a Nintendo Wii-mote controller and a Nintendo Wii balance board [18,20,29,33]. Notably, 9 of these 11 papers featuring custom-built hardware have addressed either cognitive function or physical function or activity promotion, with only 2 articles concerned with leisure. It was also noted that only 7 of these articles provided detailed information regarding the introduction, teaching, and support methods used during the intervention.

#### Software and Game Selection

A majority of the articles utilizing commercially available hardware paired it with commercially available software. Articles that chose to utilize the Nintendo Wii hardware also paired it with the Nintendo Wii software (Nintendo Co Ltd) including Nintendo Wii-Fit [25,26,32,37,38,40,41] or Nintendo Wii-Sports [25,26,28,30,31,34-38,44-47], with some articles utilizing both software packages. These software applications were used across all 3 contexts, namely, cognitive, physical, and leisure activities.

Of the articles featuring Microsoft Kinect hardware, only 2 used commercially available Kinect software (Microsoft Corp): Kinect Sports [26,36], Kinectimals [36], and Fruit Ninja [36]. The remaining studies created their own custom-built software for the Microsoft Kinect including the Kimentia software [27] and software created through a program called Unity3D (Unity Technologies) [42]. In the only study that featured the commercially available PlayStation EyeToy hardware, the corresponding EyeToy software was used, exclusively the Bubblepop game (Sony Corp) [24].

The 11 articles that created custom-built hardware also paired it with custom software programs, including unspecified commercially available software [19,21]; SketchUp (Google, Mountain View), XNA game software (Microsoft Corp), and 3Ds Max Studio software (Autodesk Inc) [18,20,29,33]; Kinempt (custom-built) [22]; Microsoft Kinect software development kit (Microsoft Corp) [43]; XaviX (SSD Co Ltd) [48]; and interactive rehabilitation exercise (IREX) software (GestureTek) [39]. The remaining study designed a graphical user interface, but made no mention of the software used for development [23]. These 11 studies tended to focus more on cognitive and physical use and provided very limited, if any information regarding the introduction, teaching, and support methods used during the activity, which were mostly delivered on an individual basis.

Four of the reviewed articles [18,20,29,33] utilized a custom-built motion-based technology platform called FitforAll, which consisted of an integrated touchscreen interface used in conjunction with the Nintendo Wii-mote and the Nintendo Wii balance board. This platform featured a physical and cognitive training program with the aim of assessing the impact of combined training on cognition and physical activity promotion in people living with dementia or MCI [18,20,29,33]. In addition, 2 studies looked at a bespoke motion-based technology music therapy program called MinWii [19,21], which was created by merging a television screen, a personal computer, and motion-based technologies such as the Nintendo Wii-mote and an infrared sensor bar. The technology allowed participants to play familiar songs or improvise a scale of their choice, and was used to promote positive self-esteem and reminiscing among people living with dementia.

### Individual or Group Use

The following section addresses the fourth research question, which explores the use of the motion-based technology for people living with dementia or MCI as a group or individual activity. Five of the studies implemented the motion-based technology in both a group and individual setting [30,37,38,42,46], and therefore these articles were counted under both categories.

#### Individual Use

Seventeen articles presented the motion-based technology to people with dementia or MCI as an individual activity (Table 1). These articles primarily addressed cognitive [22,24,29, 35,37,38,43,46,47] and physical measures [23,33,38-41,44,45], with leisure activity receiving the least amount of focus [24,30,42,46,47]. Although the reasoning behind this was not clear, it can be suggested that perhaps it is easier to track and measure cognitive and physical variables among individuals rather than groups. In addition, only 8 of these 17 articles provided all the information regarding the methods used to introduce, teach, and support people living with dementia or MCI to interact with the technology [22,29,30,33,35,42,46,47].

#### Group Use

Nineteen articles used the motion-based technology in a group setting (Table 1). Of these articles, 12 studies addressed a cognitive variable [18,21,26,28,31,32,34,36-38,45,48], 11 addressed an aspect of leisure [19,21,25,26,30,32,34,42,44-46], and 7 addressed a physical measure [20,26,30,37,38,44,45]. This suggests that using the motion-based technology in a group setting has the ability to support several aspects of well-being for people living with dementia or MCI. In addition, the research in 8 of the 13 articles addressing leisure was conducted within a group setting [19,21,25,26,32,34,44,45], with the remaining articles focusing on individual activity [24,47], or a combination of both [30,42,46]. This suggests that the group dynamic may have contributed to the positive leisure experience.

Several articles included the feedback from researchers, staff, caregivers, and participants living with dementia or MCI, reporting that the group dynamic added an extra social component to the gaming activity. Presenting the motion-based technology as a group activity was reported to promote social interaction among people with dementia and MCI [26,30,32,45,48], maintain social skills among people with dementia [34], and reduce social barriers for people with MCI [31]. The group dynamic also created an opportunity to promote teamwork and socialization [20,26,28,30-32,44,45]. It was also reported that using the motion-based technology in a group setting encouraged friendly competition [44,45] and intergenerational connections for people living with dementia [19,21,26].

### Introduction, Teaching, and Support

The fifth and the final research question aimed to identify the methods used to introduce, teach, and support people living with dementia or MCI when engaging with the motion-based technology. This is particularly important as people with dementia are indeed able to learn new things with the right support and prompting, which can help prolong independence and support a good quality of life [5]. However, this proved to be the most significant gap in the literature. Of the 31 articles included in the final review, only 19 made a reference to the way in which participants were introduced, taught, and supported to use the technology. In addition, there was major variation in the amount of information and level of detail provided.

#### Introduction Methods

Information related to the introduction of the motion-based technology with people living with dementia or MCI as discussed in the literature elsewhere has been analyzed here (Table 1). The way a technology is introduced to a person with dementia or MCI (ie, what it is, what it does, why it would be beneficial to them, and so on) is crucially important as the introduction of such devices could potentially influence the individual’s choice or desire to learn to use it [52]. In other words, introduction essentially sets the tone for teaching. For example, people with dementia or MCI are less likely to adopt a technology if they are unable to figure out what it does or how it is relevant to them [52].

Tobiasson, 2009 [44], who conducted their study as a group activity, gradually introduced participants living with dementia to the technology one at a time to give their full attention to teaching the person to handle and interact with the console system before transitioning to a group environment. Legouverneur et al, 2011 [35] provided participants with dementia or MCI with a 1-h introduction session where they were instructed to create their own virtual player (avatar) to teach them to handle and use the Nintendo Wii-mote. In addition, they suggested that the technology should be initially presented in a simple and understandable way [35]. Furthermore, Cutler et al, 2015 [26] highlighted the importance of considering the interests of people with dementia when first introducing new technology to choose activities that are relevant, meaningful, and achievable for the individual.

Weybright et al, 2010 [47] provided participants with MCI with an initial introduction session before the data collection phase where they were taught how to use the Nintendo Wii controller, how to navigate the system, and how to play the games. This approach was similar to that of Boulay et al, 2011 [21], who gave participants with dementia an introduction session to familiarize themselves with the interface. Higgins et al, 2010 [30] also provided an individual introduction session to participants with dementia or MCI prior to the study, but this was done to increase the person’s confidence and competency with the technology before transitioning into a group environment.

Chang et al, 2011 [22] introduced their gesture recognition system to a participant with dementia using verbal instructions. If the participant did not understand how to interact with the system, the researchers would provide the person with a less-intrusive prompt (verbal) followed by a more-intrusive prompt (gesture) if the user was still finding it difficult [22]. In addition, Ulbrecht et al, 2012 [46] stated that therapists, which included general nurses, geriatric nurses, or occupational therapists, received training in introducing and supporting people with dementia or MCI to engage in activities presented on the motion-based technology prior to the intervention, but they did not elaborate on what this training entailed. Through further investigation, it was found that only 9 articles [18,20,21, 30,33,34,39,45,46] used trained therapists (occupational therapists, physiotherapists, recreational therapists, psychologists, nurses, diversional therapists, and physical educators) to lead the interventions. Of further interest, many of these studies focused on an aspect of cognitive or physical function.

#### Teaching Methods

The cognitive challenges faced by people with dementia or MCI such as working memory difficulties, and impairments in attention, visuospatial abilities, and motor skills make it challenging for them to learn new information. Therefore, it is important to use teaching techniques that maximize involvement of spared abilities and avoid involvement of impaired abilities [5]. In this review, various examples of training techniques were used to teach participants to interact with the technology, illustrating the diverse and purposeful use of several techniques to accommodate the needs of people living with MCI or dementia (Table 1). For example, Leahey and Singleton, 2011 [34] mentioned about breaking the movement sequence down into small steps, which supports the conclusions of the studies [28,34,35], whose findings provide evidence of spared procedural learning capacity in people living with either dementia or MCI.

Cutler et al, 2015 [26] and Tobiasson et al, 2015 [45] both recommended providing participants with dementia initial support including verbal and physical cues, but gradually reducing these cues over time as they became more competent with the technology. In addition, both groups [26,45] suggested using positive encouragement and personalizing teaching approaches for each individual. Other training techniques for people with dementia or MCI included the use of verbal cues [35,44,45], repeated instructions [21,25,35], sounds [44], physical cues or gesture demonstrations [35,44,45], and prompts [47].

Hughes et al, 2014 [31] took a more extensive approach, providing participants with MCI with a 90-min session per week for 6 weeks to increase competence with the technology and to train participants to use the system and play the Nintendo Wii Sports games. In contrast, Chang, Chen, and Chuang, 2011 [22] designed their technology with the intent of the system training the participants, which meant that the research personnel provided no additional training after the initial introduction session. Although this study only involved 1 participant with dementia, meaning that no major conclusions can be drawn, the gesture recognition system was successful in prompting the individual through occupational tasks [22]. This approach was similar to that of Konstantinidis et al, 2016 [33], where the motion-based platform itself was designed to give instructions, prompts, praise, and guidance in order to train participants with dementia to use the technology and complete the activities.

#### Support Methods

Few articles mentioned the methods utilized to support people living with dementia or MCI during the interaction with the motion-based technology. Of interest is that verbal prompts and cues were often utilized as both teaching techniques and support methods [21,33]. In addition, movement cues and physical guidance were also offered as support methods for users by leading them through the sequence as required (eg, placing a hand over theirs) [35,44,47]. Fenney and Lee, 2010 [28] offered verbal prompts and cues to support participants with dementia during sessions as required, but otherwise they were encouraged to just play. By the end of the 9-week training, participants with dementia required significantly less prompting and were able to verbally explain and physically demonstrate the game instructions [28]. Furthermore, Siriaraya and Ang, 2014 [42] suggested the use of prompts to provide support to people living with dementia as required. This coincides with the recommendations of Tobiasson et al, 2015 [45] who suggested that support methods for people with dementia regarding the game and the movements should be personalized to each individual (ie, giving the right amount and type of support that the person requires). Whereas several articles concluded that people living with dementia or MCI require less support over time while interacting with the motion-based technology [28,30,34,35,44,45], Cutler et al, 2015 [26] suggested that after a period of initial support, people with dementia may not require any ongoing support while using such technologies.

The FitforAll platform presented by González-Palau et al, 2014 [29] and Konstantinidis et al, 2016 [33] was designed to give instructions, prompts, praise, and guidance in order to support participants with dementia to use the technology and complete the activities. With this approach, the only involvement required from the therapists was meeting with participants once in 2 weeks to determine whether to raise, decrease, or maintain the current intensity level of the platform. In contrast, Billis et al, 2011 [20], who also presented the FitforAll platform, mentioned about trained therapists or psychologists who were present at every session to offer as much support to participants with dementia or MCI as needed. The reasoning behind this variation was not clear. Similar to Billis et al, 2011 [20], Benveniste et al, 2010 [19], and Boulay et al, 2011 [21], who used a motion-based music therapy platform called MinWii, also mentioned the use of trained psychologists or therapists who were present at all sessions to offer help to participants with dementia when required. However, they did not elaborate on exactly what help was offered or when.

## Discussion

### Principal Findings

Although the current literature supports the use of the motion-based technology for people living with dementia and MCI, this area of research is still in its infancy and requires further investigation. This review highlights the potential application of this technology across several contexts to improve the well-being of people living with dementia and MCI, including cognitive, physical, and leisure activities. As previously reported with the touchscreen technology [9], the focus of research till date has been on cognitive function rather than providing meaningful leisure pastimes. This is a particularly interesting result as participants from studies across all contexts reported that the motion-based technology, and the activities presented on this type of technology were enjoyable [19-21, 24-26,30-33,39,44,45,47,48], engaging [19,20,24,25,28,30- 34,41,47,48], usable [19-21,25,26,33,41], stimulating [30,31,33], empowering [24,26,34], fun [19,25,33,44], acceptable [46], motivating [28,39], encouraging [19], meaningful [34], and perceived as a positive experience overall [35,46]. This suggests that utilizing the motion-based technology to provide meaningful leisure activities for people living with dementia or MCI warrants further exploration.

The results also demonstrate that the motion-based technology can be utilized in both group and individual settings, highlighting the potential for application in a wide range of environments including personal houses, day programs, and long-term care homes. There was a relatively even distribution between the technology use in group and individual settings, although the reason for the choice was frequently not explained. From the review it appeared that technology applications relating to cognitive and physical parameters were more often conducted as individual sessions with leisure activities in groups. This may be because the group dynamics increases opportunities for social interaction and communication, while supporting teamwork, group cohesion, friendly competition, and intergenerational connections, which are more conducive to leisure use of motion-based technologies.

The majority of studies used commercially available technologies over the bespoke ones. Cases in which custom technologies were used, they had been designed to address specific cognitive and physical variables in an individual setting. The Nintendo Wii console was the most used commercially available hardware, with the Nintendo Wii Sports and Nintendo Wii Fit games (Nintendo Co Ltd) the most used activities. Although there was less research with the more recently created Xbox Kinect console (Microsoft Corp), the Kinect has significant potential for this population as the device does not use any kind of remote or controller, allowing for a more user-friendly and natural experience based purely on organic gestures. In addition, even though these commercially available devices are not specifically designed for this population, the findings suggest that this technology can still be of benefit to people living with dementia or MCI.

It is critically important for game developers to be considerate of the cognitive and physical challenges experienced by people with dementia or MCI while creating the motion-based technology software for this population. For example, the methods used within the literature to introduce, teach, and support people with dementia or MCI to interact with the motion-based technology could be integrated into aspects of the software (ie, games) to further enhance usability, enjoyment, and increase the potential of this type of technology even more. Future recommendations include collaboration with several stakeholders including people living with dementia or MCI, researchers, nonprofit organizations, day program staff, caregivers, and game developers. It is also recommended that further work involving individuals living with dementia or MCI in the design and development process is needed to fully realize the potential of this technology.

The most prominent gap in the literature related to a lack of information about how to introduce the technology, teach people with dementia or MCI to use it, and support them in continued use. Of the 31 articles included in the final review, only 19 [19-22,25,26,28-31,33-35,42,44-48] mentioned the methods used to help people living with dementia or MCI to learn and become competent with the technology. The literature shows clear evidence that people with dementia or MCI can and are willing to learn to use the motion-based technology; therefore, it is ideal to continue to discover and develop ways in which to optimally support these individuals to facilitate learning, enjoyment, and success. Further investigation is required to produce systematic guidelines for researchers, clinicians, care providers, and families to implement this technology to benefit people living with dementia and MCI.

### Conclusions

Our findings have outlined several areas of knowledge regarding the use of the motion-based technology for people living with dementia or MCI, while highlighting gaps in the literature that warrant further investigation. With this, this review adds to the body of knowledge supporting the use of novel technologies to promote a good life for people living with dementia or MCI. It is clearly evident that people living with dementia or MCI can learn to use the motion-based technology and that this hardware can provide an avenue for cognitive stimulation, physical activity, and engaging leisure activities. However, further research regarding the introduction, teaching, and support of these individuals while using this type of technology is warranted, in addition to including their input in the development of hardware and software for this specific population.

## Abbreviations

MCI: mild cognitive impairment
NUI: natural user interface
PC: personal computer
TV: television

## References

[1] WHO.

[2] Alzheimer.ca.

[3] UN.

[4] Alzheimer.ca.

[5] Astell A (2013). Technology and fun for happy old age. Technologies for Active Aging.

[6] Alzheimer.ca.

[7] Astell AJ, Joddrell P, Groenewoud H, de Lange J, Goumans M, Cordia A, Schikhof Y (2016). Does familiarity affect the enjoyment of touchscreen games for people with dementia?. Int J Med Inform.

[8] Groenewoud H, Schikhof Y, Astell AJ, Goumans M, de Lange J (2014). iPad happy games for people with dementia as pleasant and meaningful activity. Alzheimers Dement.

[9] Joddrell P, Astell AJ (2016). Studies involving people with dementia and touchscreen technology: a literature review. JMIR Rehabil Assist Technol.

[10] Tak SH, Beck C, Hong SH (2013). Feasibility of providing computer activities for nursing home residents with dementia. Nonpharmacol Ther Dement.

[11] Anderson-Hanley C, Arciero PJ, Brickman AM, Nimon JP, Okuma N, Westen SC, Merz ME, Pence BD, Woods JA, Kramer AF, Zimmerman EA (2012). Exergaming and older adult cognition: a cluster randomized clinical trial. Am J Prev Med.

[12] Chao Y, Scherer YK, Montgomery CA (2015). Effects of using Nintendo Wii™ exergames in older adults: a review of the literature. J Aging Health.

[13] Schell R, Hausknecht S, Zhang F, Kaufman D (2015). Social benefits of playing Wii bowling for older adults. Games and Culture.

[14] Sin H, Lee G (2013). Additional virtual reality training using Xbox Kinect in stroke survivors with hemiplegia. Am J Phys Med Rehabil.

[15] Galna B, Jackson D, Schofield G, McNaney R, Webster M, Barry G, Mhiripiri D, Balaam M, Olivier P, Rochester L (2014). Retraining function in people with Parkinson's disease using the Microsoft kinect: game design and pilot testing. J Neuroeng Rehabil.

[16] Venugopalan J, Cheng C, Stokes TH, Wang MD (2013). Kinect-based rehabilitation system for patients with traumatic brain injury. Conf Proc IEEE Eng Med Biol Soc.

[17] Moher D, Liberati A, Tetzlaff J, Altman DG (2009). Preferred reporting items for systematic reviews and meta-analyses: the PRISMA statement. Ann Intern Med.

[18] Bamidis PD, Fissler P, Papageorgiou SG, Zilidou V, Konstantinidis EI, Billis AS, Romanopoulou E, Karagianni M, Beratis I, Tsapanou A, Tsilikopoulou G, Grigoriadou E, Ladas A, Kyrillidou A, Tsolaki A, Frantzidis C, Sidiropoulos E, Siountas A, Matsi S, Papatriantafyllou J, Margioti E, Nika A, Schlee W, Elbert T, Tsolaki M, Vivas AB, Kolassa IT (2015). Gains in cognition through combined cognitive and physical training: the role of training dosage and severity of neurocognitive disorder. Front Aging Neurosci.

[19] Benveniste S, Jouvelot P, Pequignot R (2010). The MINWii Project: Renarcissization of Patients Suffering from Alzheimer's Disease Through Video Game-Based Music Therapy.

[20] Billis A, Konstantinidis E, Ladas A, Tsolaki M, Pappas C, Bamidis P (2011). Evaluating affective usability experiences of an exergaming platform for seniors.

[21] Boulay M, Benveniste S, Boespflug S, Jouvelot P, Rigaud A (2011). A pilot usability study of MINWii, a music therapy game for demented patients. Technol Health Care.

[22] Chang Y, Chen S, Chuang A (2011). A gesture recognition system to transition autonomously through vocational tasks for individuals with cognitive impairments. Res Dev Disabil.

[23] Chilukoti N, Early K, Sandhu S, Riley-Doucet C, Debnath D (2007). Assistive Technology for Promoting Physical and Mental Exercise to Delay Progression of Cognitive Degeneration in Patients with Dementia.

[24] Colombo M, Marelli E, Vaccaro R, Valle E, Colombani S, Polesel E, Garolfi S, Fossi S, Guaita A (2012). Virtual reality for persons with dementia: an exergaming experience. Gerontechnology.

[25] Cutler C, Hicks B, Innes A (2014). Technology, fun and games. Journal of Dementia Care.

[26] Cutler C, Hicks B, Innes A (2015). Does digital gaming enable healthy aging for community-dwelling people with dementia?. Games and Culture.

[27] De Urturi Breton Z, Zapirain B, Zorrilla A (2012). Kimentia: Kinect based tool to help Cognitive Stimulation for individuals with Dementia.

[28] Fenney A, Lee TD (2010). Exploring spared capacity in persons with dementia: what Wii can learn. Act Adapt Aging.

[29] González-Palau F, Franco M, Bamidis P, Losada R, Parra E, Papageorgiou SG, Vivas AB (2014). The effects of a computer-based cognitive and physical training program in a healthy and mildly cognitive impaired aging sample. Aging Ment Health.

[30] Higgins HC, Horton JK, Hodgkinson BC, Muggleton SB (2010). Lessons learned: staff perceptions of the Nintendo Wii as a health promotion tool within an aged-care and disability service. Health Promot J Austr.

[31] Hughes TF, Flatt JD, Fu B, Butters MA, Chang CH, Ganguli M (2014). Interactive video gaming compared with health education in older adults with mild cognitive impairment: a feasibility study. Int J Geriatr Psychiatry.

[32] Kayali F, Luckner N, Hödl O, Fitzpatrick G, Purgathofer P, Stamm T, Schlager-Jaschky D, Mosor E (2013). Elements of play for cognitive, physical and social health in older adults. Human Factors in Computing and Informatics.

[33] Konstantinidis EI, Billis AS, Mouzakidis CA, Zilidou VI, Antoniou PE, Bamidis PD (2016). Design, implementation, and wide pilot deployment of FitForAll: an easy to use exergaming platform improving physical fitness and life quality of senior citizens. IEEE J Biomed Health Inform.

[34] Leahey A, Singleton J (2011). Utilizing therapeutic recreation to empower persons with Alzheimer's in a day center: a case report. Ther Recreation J.

[35] Legouverneur G, Pino M, Boulay M, Rigaud A (2011). Wii sports, a usability study with MCI and Alzheimer's patients. Alzheimers Dement.

[36] Liou M, Chen S, Fu H, Chiang I (2015). Effects of somatosensory video games on simple reactions of institutional-dwelling older adults with mild-cognitive impairments.

[37] McCallum S, Boletsis C (2013). A Taxonomy of Serious Games for Dementia. Games for Health: Proceedings of the 3rd European conference on gaming and playful interaction in health care.

[38] McCallum S, Boletsis C (2013). Dementia Games: A Literature Review of Dementia-Related Serious Games. Serious Games Development and Applications.

[39] McEwen D, Taillon-Hobson A, Bilodeau M, Sveistrup H, Finestone H (2014). Two-week virtual reality training for dementia: single case feasibility study. J Rehabil Res Dev.

[40] Padala KP, Padala PR, Burke WJ (2011). Wii-Fit as an adjunct for mild cognitive impairment: clinical perspectives. J Am Geriatr Soc.

[41] Padala KP, Padala PR, Malloy TR, Geske JA, Dubbert PM, Dennis RA, Garner KK, Bopp MM, Burke WJ, Sullivan DH (2012). Wii-fit for improving gait and balance in an assisted living facility: a pilot study. J Aging Res.

[42] Siriaraya P, Ang C Recreating Living Experiences from Past Memories through Virtual Worlds for People with Dementia.
2014 Presented at: Proceedings of the SIGCHI Conference on Human Factors in Computing Systems; April 26-May 1;
New York: 3977-3986.

[43] Tarnanas I, Schlee W, Tsolaki M, Müri R, Mosimann U, Nef T (2013). Ecological validity of virtual reality daily living activities screening for early dementia: longitudinal study. JMIR Serious Games.

[44] Tobiasson H (2009). Physical action gaming and fun as a tool within elderly care - Game over or play it again and again.

[45] http://www.ijdesign.org/ojs/index.php/IJDesign/article/view/1886.

[46] Ulbrecht G, Wagner D, Gräßel E (2012). Exergames and their acceptance among nursing home residents. Act Adapt Aging.

[47] Weybright E, Dattilo J, Rusch F (2010). Effects of an interactive video game (Nintendo Wii) on older women with mild cognitive impairment. Ther Recreation J.

[48] Yamaguchi H, Maki Y, Takahashi K (2011). Rehabilitation for dementia using enjoyable video-sports games. Int Psychogeriatr.

[49] Wu Y, Damnée S, Kerhervé H, Ware C, Rigaud A (2015). Bridging the digital divide in older adults: a study from an initiative to inform older adults about new technologies. Clin Interv Aging.

[50] Hicks B, Innes A, Nyman S, Cash M http://www.scie-socialcareonline.org.uk/a-how-to-guide-setting-up-and-running-gaming-technology-groups-for-people-with-dementia/r/a11G000000CTMJLIA5.

[51] Scie.org.

[52] Gibson G, Dickinson C, Brittain K, Robinson L (2015). The everyday use of assistive technology by people with dementia and their family carers: a qualitative study. BMC Geriatr.

